# A dual-capability digital portrait framework for identifying community age-friendly service needs

**DOI:** 10.1186/s12877-026-07383-0

**Published:** 2026-03-25

**Authors:** Jie Xue, Wei Guo, Guosheng Wang

**Affiliations:** Academy of Arts & Design, Tsinghua University, Beijing, 100084 China

**Keywords:** Community elderly care, Service need identification, Digital capability portrait, Decision support, Capability assessment

## Abstract

**Background:**

It is essential to enhance the effectiveness of elderly care systems by accurately identifying community age-friendly service needs. Existing need assessment approaches often rely on self-reported demands or single-dimensional capability assessments, which may fail to capture the complex interaction between older adults’ functional status and their caregiving environments.

**Methods:**

This study proposes a methodological framework for identifying community age-friendly service needs based on dual digital capability portraits. The framework integrates two analytical dimensions: individual functional capability and family caregiving capability. Firstly, a dual capability assessment framework was developed to capture the multidimensional characteristics of older adults and their household caregiving environments. Secondly, the assessment results were transformed into structured digital capability portraits to enable the standardized representation of capability information. Thirdly, capability–service need mapping matrices were constructed to establish explicit rule-based relationships between capability states and corresponding service needs. Based on these components, a hybrid decision-support approach combining rule-based reasoning and collaborative filtering was designed to generate prioritized service recommendations. A mobile application was developed to demonstrate the operational feasibility of the proposed method using a pilot sample of community-dwelling older adults.

**Results:**

The framework facilitates the systematic translation of capability assessment outcomes into structured identification of service needs. The pilot application demonstrates how digital capability portraits can be operationalized to produce personalized service recommendations within community care settings.

**Conclusions:**

This study presents a methodological framework for identifying community age-friendly service needs through dual digital capability portraits and structured capability–service mapping.

**Supplementary Information:**

The online version contains supplementary material available at 10.1186/s12877-026-07383-0.

## Introduction

With the rapid acceleration of population ageing in China, the demographic structure is undergoing a profound transition. According to the 2024 National Report on the Development of Undertakings for the Aged, the population aged 65 years and above reached 220 million by the end of 2024, accounting for 15.6% of the total population [1]. As the size of the older population continues to expand, both the volume and structure of age-friendly service needs have changed substantially [2, 3]. Concurrently, the widespread emergence of the “4–2–1” family structure has led to a persistent weakening of traditional family-based caregiving functions [4]. In response to the multifaceted challenges associated with population ageing, China has gradually established a multi-tiered elderly care system characterized by home-based care as the foundation, community services as support, institutional care as a supplement, and the integration of medical and care services. Within this system, communities play a pivotal role as a bridge between family caregiving and professional service resources, representing a critical leverage point for improving the quality of elderly care systems [5–7].

Community age-friendly services, through localized and embedded service provision, can effectively address the daily care and basic health management needs of community-dwelling older adults. These services help reduce caregiving costs, alleviate family caregiving burdens, delay functional decline, and enhance quality of life [8–11]. In recent years, there has been increasing attention to assessing community age-friendly service needs [12–14], developing service systems [15], and applying artificial intelligence technologies in community care [16]. However, in practice, community age-friendly services still commonly encounter issues of mismatch between service supply and actual needs [17–21].

In existing studies, the identification of community age-friendly service needs primarily relies on questionnaires, interviews, or practitioners’ experiential judgment. However, these approaches exhibit several limitations in practice. First, older adults often have difficulty accurately and comprehensively articulating their service needs due to constraints related to physical function, cognitive ability, educational background, and communication capability, particularly concerning latent needs and potential risk-related demands [22]. Second, much of the existing research focuses primarily on descriptive statistics of expressed needs, while insufficient attention is given to the underlying influences of individual functional capability and family caregiving capability on the formation of service needs [23, 24]. Third, the conventional age-friendly service design pathway of “need identification–user portrait–service provision” typically constructs user portrait only after the specific needs has been identified. This approach hinders the systematic capture of older adults’ overall functional status and caregiving context, thereby constraining the precision and sustainability of service matching [25, 26].

Although prior research on community age-friendly service has made significant progress in developing service methods, notable gaps remain in identifying service needs. Specifically, existing approaches often rely on subjective self-reporting or single-dimensional assessments, limiting their ability to comprehensively reflect actual functional status and caregiving contexts of older adults. Furthermore, the moderating role of family caregiving capability in shaping service needs has received insufficient attention, resulting in a lack of evidence to guide judgments regarding the appropriate intensity of service interventions. Moreover, there is a lack of structured methods for directly translating capability assessment results into actionable service decisions, undermining the precision and consistency of identifying community age-friendly service needs.

To address these limitations, this study proposes a methodological framework for identifying community age-friendly service needs based on dual digital capability portraits. The framework integrates individual functional capability and family caregiving capability to assess service needs within a broader care context. First, a dual capability assessment framework is constructed to capture the multidimensional characteristics of older adults and their caregiving environments. Second, the assessment outcomes are transformed into digital capability portraits to enable standardized representation of capability information. Third, capability–service need mapping matrices are established to define explicit relationships between capability states and service needs. Finally, a hybrid decision-support mechanism, which combines rule-based reasoning and collaborative filtering, is introduced to generate prioritized service recommendations. A pilot implementation involving 21 participants was conducted as a proof-of-concept demonstration of the operational feasibility of the proposed framework.

## Methods

**Fig. 1:**
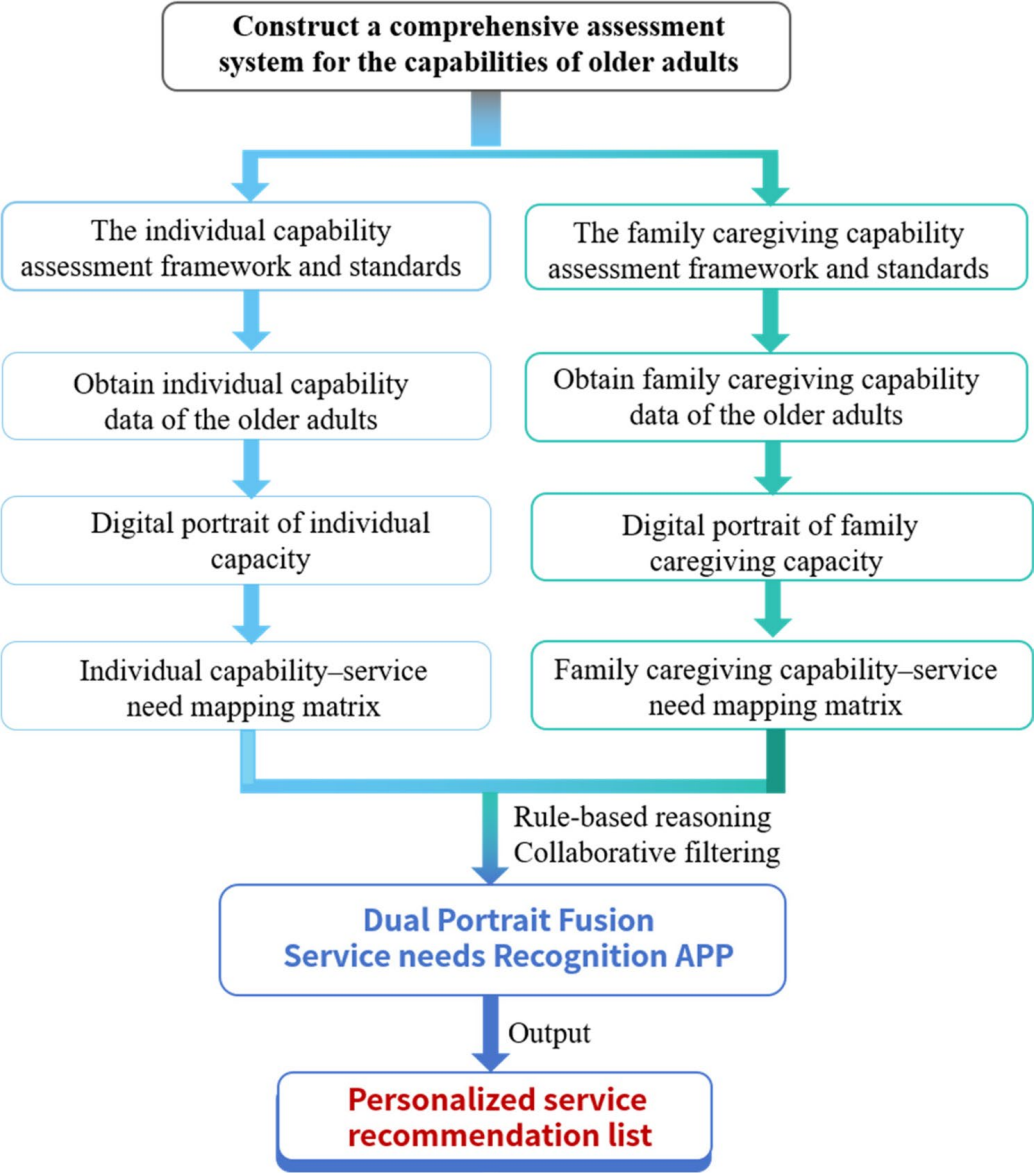
Framework diagram of community age-friendly service needs identification method based on dual digital capability portraits

This study proposes a methodological framework for identifying the community age-friendly service needs based on dual digital capability portraits, as illustrated in Fig. 1. The framework comprises three primary components: (1) a dual capability assessment framework that evaluates individual functional capability and family caregiving capability; (2) digital capability portraits that represent structured capability information; and (3) capability–service need mapping matrices that translate capability states into potential service interventions. The methodological workflow encompasses capability assessment, digital capability portrait construction, capability–service mapping, and the generation of service recommendations. The pilot implementation, involving 21 community-dwelling older adults, was conducted solely to demonstrate the operational feasibility of the framework and should be interpreted as a proof-of-concept demonstration rather than as empirical outcome validation.

### Construction of the dual capability assessment framework

Previous studies have demonstrated the feasibility and practical value of applying structured and simplified comprehensive geriatric assessment tools in primary care and community settings, providing an important reference for the development of community-oriented capability assessment frameworks [27]. Building on this evidence, the present study constructs a dual capability assessment framework encompassing both individual functional capability and family caregiving capability.

The individual capability assessment framework utilized in this study was developed by integrating concepts from the International Classification of Functioning, Disability and Health (ICF) [28] and the Chinese national standard Specification for Assessment of the Capability of the Elderly (GB/T 42195–2022) [29]. The ICF framework provides a comprehensive conceptual structure for describing functional health and disability across physical, cognitive, psychological, and social domains. Meanwhile, GB/T 42,195–2022 offers a standardized assessment tool that is widely applied in elderly care management and long-term care evaluation in China. Rather than directly adopting these frameworks, the present study adapted and operationalized relevant capability indicators to better align with the requirements for identifying community-based age-friendly services. Indicators from GB/T 42,195–2022 related to activities of daily living, mobility, cognitive orientation, communication ability, and health status were incorporated as the primary reference structure. Additionally, the broader functional perspective of the ICF framework was employed to ensure that multiple dimensions of functioning were represented in the assessment system.

Several adjustments were made to adapt the framework to the context of community age-friendly service planning. First, indicators were selected and simplified to enhance feasibility in community assessment settings while retaining essential functional dimensions. Second, indicators reflecting community participation and modern living conditions—such as social interaction and digital literacy—were incorporated to capture capability domains increasingly relevant to older adults living in digitally connected communities. Third, all indicators were standardized using a unified five-point ordinal scale to facilitate structured representation in digital capability portraits and subsequent computational analysis. Through this process, a multidimensional capability assessment framework consisting of 28 indicators was constructed, providing a foundational data structure for digital capability portraits and subsequent service need identification. The detailed scoring criteria for each indicator are provided in Supplementary File 1 to enhance transparency and reproducibility of the assessment process.

In community-based elderly care systems, where home-based care remains the primary mode of support, the capability of family caregiving plays a critical role in shaping older adults’ reliance on external services. Previous studies consistently demonstrate that the availability of family caregiving resources, such as financial support, caregiving time, caregiving skills, and emotional support, significantly influences the demand for formal care services among community-dwelling older adults [30–35]. Therefore, assessing the caregiving capability of households provides an important complementary perspective for understanding the emergence of community service needs.

The conceptual design of the family caregiving capability framework in this study is informed by research on informal caregiving systems, household support structures in long-term care, and existing assessment practices related to family care resources. In particular, prior studies on long-term care evaluation and family support assessment emphasize the multidimensional nature of caregiving capability, which includes economic resources, practical caregiving capacity, and emotional support within the household. These dimensions serve as the conceptual basis for constructing the framework.

The assessment of individual capability primarily focuses on the functional status of older adults, encompassing activities of daily living, cognitive function, and mobility. In contrast, the assessment of family caregiving capability evaluates the resources available at the household level to support the older adult. In essence, individual capability reflects the care needs arising from functional limitations, whereas family caregiving capability indicates the extent to which these needs can be met within the household. The interplay between these two dimensions serves as the analytical foundation for the dual-capability framework proposed in this study. In this analytical process, individual functional capability represents the primary source of potential service needs, while family caregiving capability functions as a moderating factor that determines whether these needs can be addressed within the household or necessitate external community service intervention.

### Construction of digital capability portraits

Based on the capability assessment data, digital capability portraits were constructed separately for individual capabilities and family caregiving capabilities of older adults. These digital portraits encode multidimensional capability characteristics in vector form, enabling a standardized digital representation of older adults’ capability structures. Through digital capability portraits, both individual-level capability patterns and population-level capability distribution characteristics within a community can be analyzed. Serving as the core input variables for the subsequent service need identification method, digital capability portraits make assessment outcomes computable and reusable, thereby providing a solid data foundation for service need identification and hierarchical recommendations.

### Construction of capability–service need mapping matrices

To enable structured mapping from capability states to service needs, this study constructed two mapping matrices: an individual capability–service need matrix and a family caregiving capability–service need matrix. These matrices delineate potential types of community age-friendly service needs and corresponding levels of intervention intensity across varying capability states. The capability–service need mapping matrices were developed based on existing research evidence, community age-friendly service practices, and the internal logic of capability assessment. A rule-based approach was employed to explicitly define the correspondence between capability indicators and service items. Serving as a core rule foundation for service need identification, the matrices ensure interpretability and consistency in the decision-making process.

### Service need identification and recommendation method

Based on the dual digital capability portraits and the capability–service need mapping matrices, a method for identifying service needs and providing hierarchical recommendations has been further developed. This method takes into account both the degree of individual capability impairment and the level of family caregiving support. By integrating rule-based reasoning with collaborative filtering techniques, the service needs are effectively identified, prioritized, and stratified. The method produces a structured list of community age-friendly service needs, which includes service categories, recommended priority levels, and intervention intensity grades, thereby enhancing the systematic nature and consistency of service need identification.

### Application implementation and pilot demonstration

To demonstrate the feasibility of the proposed method, an application tool was developed for identifying community age-friendly service needs, based on the aforementioned framework and methodology. This tool facilitates the entry of capability assessment data, automates the identification of service needs, thereby providing auxiliary support for service planning and resource allocation in community elderly care practices. The application example aims to illustrate the operational workflow of the proposed method and is not intended to evaluate the effectiveness of specific service interventions.

## Results

The results presented in this section primarily pertain to the intermediate outputs of method development, rather than to statistical results derived from intervention studies or experimental designs.

### Individual capability assessment framework and standards

By incorporating the characteristics of community age-friendly service needs, an individual functional capability assessment framework has been developed, as illustrated in Table 1. The framework consists of 28 secondary indicators, all assessed using a unified five-point ordinal scale ranging from 0 to 4. This structure enables a comprehensive and sensitive reflection of older adults’ functional status across physical, cognitive, emotional, and social domains, thereby providing a standardized basis for subsequent identification of service needs.

**Table 1:** Evaluation system for the individual capabilities of older adults

Domain	Indicator	Brief description
Basic ADL	Eating	Ability to eat independently and swallow safely
	Personal grooming	Washing face, brushing teeth, grooming, nail care
	Bathing	Washing and drying the body
	Dressing (upper body)	Dressing/undressing upper-body clothing
	Dressing (lower body & footwear)	Dressing/undressing trousers, shoes, and socks
Continence & toileting	Urinary continence	Ability to control and empty the bladder
	Bowel continence	Ability to control and evacuate stool
	Toileting	Using the toilet and performing personal hygiene
Mobility	Bed mobility	Turning in bed; sitting up and lying down
	Bed–chair transfer	Transfers between sitting and standing
	Walking on level ground	Ambulation on flat surfaces
	Stair climbing	Ascending and descending stairs
Cognitive orientation	Time orientation	Awareness of time (year, month, date, day/night)
	Spatial orientation	Awareness of location and surroundings
	Person orientation	Recognition of familiar people
Cognitive function	Memory	Short-term, recent, and remote memory
Communication	Comprehension	Understanding verbal and non-verbal information
	Expression	Ability to express needs and ideas
Behavioral & emotional status	Aggressive behavior	Physical or verbal aggressive behaviors
	Depressive symptoms	Presence of depressive or negative emotional symptoms
Consciousness & sensory function	Level of consciousness	Responsiveness to self and environment
	Vision	Visual perception under best-corrected vision
	Hearing	Ability to perceive and understand sounds
Instrumental & social function	Managing daily affairs	Planning and completing daily tasks (e.g., medication management)
	Using transportation	Independent use of transportation
	Social interaction	Participation in social life and appropriate interaction
	Digital literacy	Use of digital devices for daily activities
Health status	Chronic disease status	Burden and control of chronic diseases

Detailed scoring criteria for each level (0–4) are provided in Supplementary File 1

The proposed assessment framework demonstrates three main advantages. First, it offers comprehensiveness, as the integration of multidimensional indicators helps to avoid biases in service need identification that may arise from single-domain assessments. Second, it ensures scientific rigor, with clearly defined indicators and scoring criteria that exhibit good reliability and validity, aligning with policy instruments such as long-term care insurance assessments. Third, it shows strong operability, as assessment items are based on observable daily behaviors with a clear implementation process, making the framework suitable for large-scale application in community elderly care settings.

### Digital portrait of individual capability

Based on the aforementioned assessment framework, standardized and quantitative capability assessments can be conducted for each older adult within the community. The individual capability characteristics can be digitally represented as a one-dimensional vector. This representation aims to formalize the structure of functional capability without engaging in parameter estimation or statistical inference. Let the digital portrait of individual functional capability for the *ith* older adult be defined as follows:

1 $$\:{A}_{i,28}=({a}_{i,1},{a}_{i,2},\dots\:,{a}_{i,j}{,\dots\:,a}_{i,28})$$

where $\:{a}_{i,j}\:$denotes the quantified score of the *ith* older adult on the *jth* capability indicator. The 28 indicators correspond to essential functional domains, which include activities of daily living, self-care ability, basic mobility, sensory function, cognitive status, psychological status, health management capability, social participation and so on.

For example, the digital capability portrait of the first older adult can be expressed as:

2 $$\:{A}_{\mathrm{1,28}}=(\mathrm{1,2},\mathrm{1,1},\mathrm{1,2},\mathrm{2,2},\mathrm{1,1},\mathrm{2,1},\mathrm{2,2},\mathrm{3,2},\mathrm{2,2},\mathrm{1,2},\mathrm{1,2},\mathrm{2,2},\mathrm{1,2},\mathrm{3,2})$$

Similarly, the digital capability portrait of the 100*th* older adult is represented as:

3 $$\:{A}_{\mathrm{100,28}}=(\mathrm{2,1},\mathrm{3,1},\mathrm{2,1},\mathrm{1,1},\mathrm{3,2},\mathrm{1,3},\mathrm{0,4},\mathrm{1,0},\mathrm{2,1},\mathrm{1,2},\mathrm{1,1},\mathrm{3,1},\mathrm{1,1},\mathrm{1,1})$$

By aggregating the digital capability vectors of all older adults in the community, an individual functional capability data matrix for the entire community can be constructed as:

4 $$\:\boldsymbol{A}=\left(\begin{array}{c}{A}_{\mathrm{1,28}}\\\vdots\\{A}_{N,28}\end{array}\right)$$

where N represents the total number of older adults in the community. This capability data matrix enables a systematic and quantitative representation of the functional capability structure of community-dwelling older adults and has multiple practical applications. First, it facilitates the identification of the distribution and heterogeneity of functional capabilities of older adults within the community, thereby supporting the detection of potentially high-risk groups. Second, it provides a data foundation for inferring the overall community-level age-friendly service needs, facilitating a shift in service planning from experience-based judgment to data-informed decision-making. Third, it offers a computable basis for the allocation of community service resources, such as caregiving personnel, rehabilitation resources, and assistive devices. Finally, it establishes a data foundation for the subsequent monitoring of capability changes and the evaluation of service-related outcomes.

### Individual capability–service need mapping matrix

The decline in individual functional capability is a fundamental factor driving the emergence of age-friendly service needs among older adults. Thus, the structured identification of capability impairments is a critical prerequisite for achieving precise service matching. Based on this premise, this study developed an individual capability–service need mapping matrix to characterize the types and intensity levels of service needs corresponding to different capability states.

During the development of the matrix, existing research evidence and practical experiences from community elderly care were integrated. Additionally, general distribution patterns of capability assessment data were taken into account. Service needs associated with various capability states were summarized using a rule-based approach, while service utilization characteristics observed among different groups of older adults were analyzed to infer the service needs structure corresponding to each capability state. By establishing structured correspondences between graded capability indicator scores and specific service needs categories, an individual capability–service need mapping matrix was formed (a schematic example is presented in Table 2).

**Table 2:** Individual capability–service need mapping matrix (Illustrative Examples)

No.	Capability	Service needs corresponding to capability score				
		Score 4	Score 3	Score 2	Score 1	Score 0
1	Eating	Anti-slip placemats; heat-resistant tableware; lightweight age-friendly utensils	Eating reminders; meal arrangement assistance; assistive utensils (e.g., anti-tremor or angled spoons)	Minor feeding assistance; swallowing training; soft or semi-liquid diet	Full feeding assistance; swallowing rehabilitation; aspiration prevention care	Feeding services; tube feeding care; nutritional support; aspiration risk management
2	Personal grooming (washing face, brushing teeth, hair grooming)	Age-friendly washing environment; anti-slip mats	Grooming reminders; easy-grip toiletry items	Assistance with face washing, hair grooming, and tooth brushing; basic nail care	Comprehensive grooming assistance; skin moisturizing care	Full grooming care; oral care with sponge swabs; intensive skin care
3	Bathing	Anti-slip flooring; grab bars; shower chairs; handheld showers	Bathing reminders; optimized placement of bathing supplies	Partial bathing assistance (upper body/back); warmth and slip prevention	Full bathing assistance; safe assistance for entering and exiting the bath	Bed bathing; assisted hair washing; pressure injury prevention

This table employs eating, personal grooming, and bathing as illustrative examples to demonstrate the mapping logic between various levels of individual capability scores and the corresponding types and intensities of community age-friendly service needs. A detailed individual capability–service need mapping matrix is provided in Supplementary Table S1. It is important to note that the table serves solely for methodological illustration and does not constitute an exhaustive list of service items

This matrix facilitates a standardized transformation from “capability assessment results” to “service need identification”, ensuring that judgments regarding service needs are no longer reliant on subjective experiences. Instead, they are derived from interpretable and reproducible inferences based on capability states. In practical applications, data from capability assessments can be further integrated into clustering analyses to continuously refine and optimize the content of the mapping matrix. Overall, the individual capability–service need mapping matrix serves as a crucial tool for identifying age-friendly service needs and as a foundational methodological component for achieving precision in the provision of community age-friendly services. This matrix provides scientific support for individualized service recommendations, community-level service planning, and policy-level resource allocation, while also establishing a methodological basis for the subsequent integration of family caregiving capabilities and intelligent algorithms.

### Family caregiving capability assessment framework and standards

The integrated assessment framework for family caregiving capability has been developed, encompassing three primary dimensions: economic support capability, caregiving capability, and emotional support capability [36]. Eight secondary indicators have been defined under these dimensions, each assessed using a standardized five-point ordinal scale ranging from 0 to 4, as detailed in Table 3.

**Table 3:** Assessment system for family caregiving capability of older adults

Domain	Indicator	Brief description
Economic capacity	Financial resources	Relative level of older adults’ monetary assets compared with the local population
	Medical expenditure burden	Proportion of older adults’ medical expenditure in total household consumption
	Housing conditions	Relative housing conditions compared with the local population
Caregiving capacity	Daily caregiving time	Average daily time that family members can provide care
	Caregiving skills	Types of caregiving skills possessed by family caregivers
Emotional support capacity	Communication and interaction	Frequency and intensity of effective communication between family members and older adults
	Participation in decision-making	Degree of older adults’ participation in family decision-making
	Family conflict level	Frequency and severity of family conflicts

Detailed scoring criteria for each level (0–4) are provided in Supplementary File 2

Economic support capability evaluates household material resources and the burden of medical expenditures, reflecting the financial sustainability of long-term caregiving. Caregiving capability assesses the time available for caregiving from family members and their caregiving skills, capturing the family’s practical ability to provide routine care and respond to emergencies. Emotional support capability evaluates the family’s ability to offer psychological and emotional support to older adults, focusing on communication and interaction, participation in decision-making, and the stability of family relationships.

In contrast to previous studies that primarily examined economic status or isolated caregiving elements, the proposed assessment framework offers several advantages. First, it provides multidimensional integration, allowing for a more comprehensive representation of overall family caregiving capability. Second, it ensures standardization and quantifiability, with clearly defined scoring criteria that facilitate implementation and comparison at the community level. Third, it is practice-oriented, as the assessment results can be directly applied to graded community service management and resource allocation decisions.

### Digital portrait of family caregiving capability

Based on the family caregiving capability assessment framework, the caregiving capability of older adults’ households can be quantitatively assessed and represented in a digital vector format. Consequently, the resulting representation forms the digital portrait of family caregiving capability.

The digital portrait of family caregiving capability for the *ith* older adult is defined as:

5 $$\:{B}_{i,8}\:=\:({b}_{i,1},\:{b}_{i,2},\:\dots\:,{b}_{i,j},\dots\:\:,{b}_{i,8})$$

where $\:{b}_{i,j}$ denotes the quantified score of the *ith* older adult on the *j*th family caregiving capability indicator. These eight indicators reflect family caregiving capabilities across various domains. For instance, the digital portrait of family caregiving capability of the first older adult can be expressed as follows:

6 $$\:{B}_{1,8}\:=\:(2,\:3,\:1,\:1,\:3,\:1,\:2,\:2)$$

Similarly, the digital portrait of family caregiving capability of the *100th* older adult is represented as:

7 $$\:{B}_{\mathrm{100,8}}\:=\:(\mathrm{3,2},\:1,\:2,\:2,\:2,\:1,\:2)$$

By vertically aggregating the family caregiving capability vectors of all older adults within the community, a comprehensive community-level data matrix of family caregiving capabilities can be constructed as:

8 $$\:\boldsymbol{B}=\left(\begin{array}{c}{B}_{\mathrm{1,8}}\\\vdots\\{B}_{N,8}\end{array}\right)$$

where N denotes the total number of older adults in the community. This family caregiving capability data matrix offers a comprehensive quantitative representation of the structure of household caregiving resources within the community. It serves as a foundational data source for analyzing variations in family caregiving capability, identifying households at heightened risk for caregiving, and developing differentiated service strategies.

### Family caregiving capability–service need mapping matrix

Family caregiving capability serves as a significant moderating factor in the formation of community service needs among older adults. Generally, a weaker family caregiving capability correlates with a greater reliance on community age-friendly services, which is accompanied by more pronounced types and higher intensity of service needs. Building upon the assessment and digital profiling of family caregiving capability, this study further constructs a family caregiving capability–service need mapping matrix to characterize external service needs patterns under varying family capability states.

In developing the matrix, the fundamental logic that “declining family caregiving capability corresponds to increasing external service needs” was adhered to. Different score levels of family caregiving capability indicators were systematically mapped to corresponding types of community services. For instance, when available family caregiving time is insufficient, older adults demonstrate an increased demand for home-based care, day care, or respite services. When family caregiving skills are limited, the demand for professional nursing, rehabilitation guidance, and health management services becomes more pronounced. Additionally, when family emotional support capability is weak, older adults exhibit a greater reliance on psychological support and social companionship services. Through a systematic examination of the relationships between family caregiving capability indicators and service needs, a family caregiving capability–service need mapping matrix was established (Table 4).

**Table 4:** Family caregiving capability–service need mapping matrix (Illustrative Examples)

No.	capability indicator	Service needs corresponding to capability score				
		Score 4	Score 3	Score 2	Score 1	Score 0
1	Financial resources	Financial management advice; optimization of expenditure structure; affordability of market-based services	Guidance on subsidy applications; intra-family financial support; expense planning	Linkage to public or low-cost services; assistance with government subsidy applications	Basic living allowances; long-term care insurance application; monitoring of financial hardship	Minimum living guarantee; government safety-net support; medical assistance; emergency financial aid
2	Medical expenditure burden	Purchase of commercial health insurance	Guidance on health insurance reimbursement procedures	Monitoring of medical expenditures; assistance with reimbursement processes	Medical assistance applications; support with long-term care insurance claims	Medical aid; catastrophic health expenditure coverage; support from major illness funds
3	Housing conditions	Safety inspection; minor improvements (e.g., anti-slip measures, lighting)	Guidance on age-friendly adaptations (e.g., grab bars, lighting, anti-slip flooring)	Partial age-friendly renovations (e.g., bathroom or bedroom)	Comprehensive age-friendly renovation plans; fall risk management	Full-scale age-friendly renovation; emergency alarm systems; intelligent monitoring and fall-prevention systems
4	Daily caregiving time	Stable routine emotional interaction	Scheduled check-ins	Home-based care services	Half-day or day-care services	24-hour professional caregiving
5	Caregiving skills	Experience sharing and peer learning	Training in emergency response or cognitive care	Training in medical, emergency, and cognitive caregiving skills	Partial training in basic, medical, emergency, and cognitive caregiving	Comprehensive training in basic, medical, emergency, and cognitive caregiving skills
6	Emotional communication and interaction	Maintenance of appropriate family emotional interaction	Regular emotional interaction; mental health reminders	Social engagement promotion; interest-based activity guidance	Psychological comfort services; companionship visits	Psychological counseling; emotional crisis intervention; loneliness risk management
7	Participation in decision-making	Active participation in decisions within personal capability	Facilitation of family consultation	Guidance on participation in activities	Emotional support	Interventions to restore decision-making rights; social integration services; family mediation
8	Family conflict level	Maintenance of a harmonious family environment	Conflict adjustment guidance; family meeting facilitation	Emotional management; conflict mediation	Family psychological counseling; stress intervention	Crisis intervention; family relationship repair; psychological treatment; safety risk protection

This table illustrates the relationship between varying levels of family caregiving capabilities and the corresponding types and intensities of community age-friendly service needs. It serves as a foundational rule-based framework for integrating family caregiving capabilities into the identification of service needs and is intended for methodological illustration rather than as a comprehensive service catalog

This matrix facilitates a standardized transformation from “family capability states” to “service intervention needs”, establishing a methodological foundation for the subsequent integration of family caregiving capability with individual functional capabilities in the comprehensive identification of service needs. It enables a structured analysis of how household-level caregiving resources influence patterns of service needs.

The family caregiving capability–service need mapping matrix, in conjunction with the individual capability–service need mapping matrix, forms a dual capability foundation for identifying community age-friendly service needs. By incorporating family caregiving capability into the analytical framework, the proposed approach more accurately reflects the real-world caregiving contexts of older adults, thereby helping to avoid both overestimations and underestimations of service needs that may occur when relying solely on individual capability assessments.

### Community age-friendly service need identification based on digital capability portraits

This study further proposes a method for identifying community age-friendly service needs based on the integration of these dual capability portraits. The proposed method utilizes the degree of individual functional capability impairment and the level of family caregiving support as complementary decision criteria, achieving precise identification and hierarchical recommendation of community age-friendly services through the following steps.

1. Rule-based capability–service mapping.

The first stage of the service identification process involves a rule-based mapping procedure. In this stage, individual capability indicators and family caregiving capability indicators are evaluated using a predefined assessment framework. The resulting capability scores are then translated into corresponding service needs utilizing the capability–service need mapping matrices developed in this study. These matrices establish deterministic relationships between capability states and potential service interventions. For instance, specific thresholds of functional limitations or caregiving insufficiencies trigger predefined service categories, such as daily living assistance, rehabilitation support, or community monitoring services. Consequently, the rule-based mapping stage ensures that all capability-derived service needs are systematically identified in a transparent and reproducible manner. This stage serves as the primary mechanism for identifying potential service needs, generating an initial list of candidate services associated with each digital capability portrait.

(2) Recommendation refinement through similarity-based analysis.

Following the rule-based identification stage, a similarity-based recommendation mechanism is employed to refine the prioritization of service needs. This component utilizes a user-based collaborative filtering approach to analyze the similarities between the digital capability portraits of various older adults. By identifying individuals with comparable capability profiles, the system can detect patterns in service needs among similar users. These similarity relationships are then leveraged to adjust the ranking of candidate services generated by the rule-based mapping stage. Thus, collaborative filtering does not replace rule-based mapping; rather, it serves as a recommendation refinement layer that enhances the prioritization of services. This two-stage design ensures both analytical transparency and recommendation adaptability. The rule-based component guarantees interpretability and deterministic need identification, while the similarity-based recommendation component introduces data-driven refinement based on patterns observed across comparable users. In summary, the rule-based mapping matrices determine which services are potentially required, while the recommendation mechanism prioritizes these services for each individual case. It should be pointed out that the collaborative filtering component is currently implemented as a prototype for demonstration purposes, and full predictive optimization using larger datasets is planned for future work.

### Application implementation for personalized identification of community age-friendly service needs

To demonstrate the feasibility and practical applicability of the proposed method, this study developed a mobile application (APP) aimed at the precise identification of community age-friendly service needs based on the aforementioned service need identification method. The application consists of the primary functional modules of capability assessment data entry, service need identification and recommendation.

To demonstrate the operational feasibility of the proposed framework, a pilot implementation was conducted involving 21 community-dwelling older adults. Participants were recruited through voluntary participation from a local community elderly service center that provides routine community-based care services for older residents. The recruitment specifically targeted older adults capable of participating in capability assessments and who provided informed consent. Capability assessments were conducted by trained researchers familiar with community elderly care assessment procedures. Data collection was performed through structured face-to-face interviews supplemented by observational evaluations of daily functioning. The assessment process adhered to the standardized individual capability and family caregiving capability frameworks proposed in this study.

The primary assessment instruments consisted of the 28-indicator individual capability assessment framework and the 8-indicator family caregiving capability assessment framework developed for this study. Each assessment required approximately 40–50 min to complete. During the assessment process, basic demographic information was also collected to provide contextual data for interpreting the capability profiles. These variables included age, gender, living arrangement, and general health status of the participants. It is important to note that the purpose of this pilot implementation was not to evaluate the effectiveness of the service recommendations but rather to demonstrate the operational feasibility of the proposed method and illustrate how digital capability portraits can be translated into structured service need outputs.

**Fig. 2:**
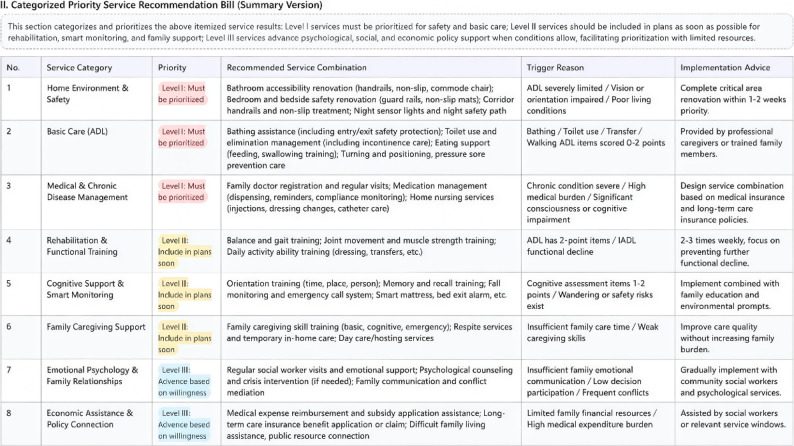
Screenshot of the service recommendation list generated by the application

For example, after the digital portraits of Mr. Zhang, A_28_=(1,1,1,1,2,2,1,1,1,2,1,2,3,3,1,1,1,1,2,1,1,3,2,1,2,1,1,1) and B_8_=(0,2,1,1,1,1,1,2) are input in the APP, a personalized list of community age-friendly service needs can be instantly generated and ranked according to priority levels, the screenshot is provided in Fig. 2. The proposed method establishes a systematic linkage between capability assessment and service decision-making. This approach provides a replicable and scalable technical pathway for the precise provision of community age-friendly services.

## Discussion

This study addresses the critical issue of identifying community age-friendly service needs by proposing a precision identification method based on dual digital portraits of older adults’ capabilities. By integrating individual functional capabilities and family caregiving capabilities within a unified analytical framework, and incorporating capability-service need mapping matrices along with a rule-driven decision-support method, the proposed approach facilitates a systematic transformation from capability assessment outcomes to structured service need information. The findings indicate that this method can support the identification and stratification of multidimensional care needs in complex community care contexts, thereby providing methodological support for decision-making in community age-friendly services.

### Main findings and methodological implications

Compared to existing needs assessment approaches, this study presents three primary methodological contributions that underscore its originality. First, the proposed framework introduces a dual-capability perspective by integrating both individual functional capability and family caregiving capability into a unified analytical structure. This dual-capability model, in contrast to conventional approaches that focus solely on individual functional status, more accurately reflects the real-world care environment in which service needs arise. Second, the study constructs explicit capability-service need mapping matrices that translate capability assessment outcomes into structured service decision rules, thereby providing a transparent pathway from assessment to service identification. Third, the framework combines rule-based reasoning with similarity-based recommendation techniques to facilitate the hierarchical prioritization of service needs. Collectively, these components establish a systematic methodological pathway for transforming multidimensional capability assessment data into actionable decision-support information for community elderly care.

Although the present framework was developed within the context of China’s community-based elderly care system, its methodological principles are broadly applicable to other aging societies. The core elements of the framework—digital capability portraits, dual-capability assessment, and capability-service need mapping—are conceptually transferable across various health and social care systems. However, the specific service taxonomies and policy references used in the mapping matrices may require adaptation to reflect local welfare systems, service infrastructures, and cultural expectations regarding family caregiving. With appropriate contextual adjustments, the framework has the potential to support data-informed service planning in diverse international settings.

### Comparison with existing studies

Although previous studies have introduced multidimensional assessment tools, such as simplified geriatric assessment instruments applied in community and primary care settings, most have remained focused on identifying high-need individuals or conducting risk screening, without systematically translating assessment results into subsequent service decision pathways or intervention strategies [27]. The present study advances existing research in several ways. First, it explicitly distinguishes between capability states and service needs, constructing a capability-driven logic for generating service needs. Second, by introducing capability–service need mapping matrices, it facilitates structured mapping from assessment outcomes to service types and intervention intensity. Third, the proposed method is embedded within a real-world community elderly care application context, demonstrating its operational feasibility. These features enhance the proposed approach’s alignment with actual decision-making processes in community elderly care practice.

### Implications for community age-friendly service practice

Recent studies on community-based care services have revealed disparities in perceived service quality and accessibility across various populations and regions. This underscores the importance of accurately identifying service needs and allocating resources based on objective capability assessments [37]. The method proposed in this study offers several practical implications for the provision of community age-friendly services. First, it provides community practitioners with a systematic tool for identifying service needs, thereby reducing reliance on individual experiential judgment and enhancing the standardization and consistency of assessment processes. Second, by incorporating family caregiving capabilities into the analytical framework, the method facilitates more effective coordination between household caregiving resources and community service provision, thereby supporting the development of differentiated and tiered intervention strategies. Third, service need lists generated from digital capability portraits can inform community-level service planning, assist in identifying priority populations, and guide resource allocation, ultimately improving the responsiveness and efficiency of community age-friendly services.

In terms of practical implementation, the proposed method can be seamlessly integrated into routine assessments for elderly care within communities. The capability assessment generally takes approximately 40 to 50 min to complete and can be efficiently conducted by trained community health workers or social service practitioners. Minimal technical infrastructure is required, consisting primarily of a digital data entry interface and a basic service recommendation module. Future implementations could see the system integrated with electronic health record platforms or community service management systems, facilitating automated identification of service needs and supporting coordinated care planning.

### Limitations and future research directions

Several limitations must be acknowledged. First, the capability-service need mapping matrices are primarily constructed using rule-based logic, which is informed by both literature and practical considerations. This approach may introduce a degree of subjectivity into the mapping relationships. Second, although standardized scoring criteria were established for the capability assessment indicators, the assessment process may still involve subjective judgments by assessors. This highlights the importance of evaluating inter-rater reliability in future research. Third, the current study utilized a relatively small pilot sample to demonstrate the feasibility of the framework. Larger datasets will be necessary to evaluate the predictive performance and scalability of the recommendation system. Finally, as the framework incorporates algorithmic recommendation components, potential algorithmic bias risks should be carefully considered in future implementations, especially when expanding the system to more diverse populations. Continuous evaluation and iterative model refinement will be essential to ensure the fairness and robustness of service recommendations.

The proposed framework represents a methodological approach for translating capability assessments into structured service need identification. Future research is needed to further validate and refine the framework through several complementary pathways. First, expert consensus methods, such as Delphi panels involving gerontology researchers, community care practitioners, and policy experts, could be utilized to evaluate and enhance the capability–service need mapping matrices. Such consensus processes would strengthen both the conceptual validity and practical relevance of the rule-based mapping relationships. Second, empirical validation studies should investigate the correlation between the framework-generated service recommendations and actual care outcomes, including service utilization, functional improvement, and quality-of-life indicators among older adults. Evaluating the predictive accuracy of the framework in identifying appropriate services would provide stronger evidence of its practical effectiveness. Third, future research should assess the reliability of the capability assessment process, encompassing both inter-rater reliability among different assessors and test–retest reliability over repeated assessments. Establishing measurement reliability is essential to ensure the consistency of the digital capability portraits used in the recommendation system. Finally, the scalability and generalizability of the framework should be examined using larger and more diverse populations across various community settings. Larger datasets would also facilitate the further development of the collaborative filtering component and enable more robust data-driven optimization of service recommendations. Collectively, these validation pathways offer a roadmap for future research aimed at enhancing the methodological robustness and practical applicability of the proposed framework.

## Conclusions

By constructing digital capability portraits of older adults and capability–service need mapping matrices, a method for identifying community age-friendly service needs is proposed. The findings indicate that the proposed approach facilitates the systematic identification and hierarchical prioritization of community age-friendly service needs, while offering interpretable decision support for community practitioners. By reducing reliance on subjective experiential judgment, this method enhances the accuracy and consistency of service need identification, providing a methodological foundation for coordinating family caregiving resources with community service provision.

## Supplementary Information

Supplementary Material 1.

Supplementary Material 2.

Supplementary Material 3.

## Data Availability

The datasets used or analyzed during the current study are available from the corresponding author on reasonable request.
