# Supplementary material for: A dual-capability digital portrait framework for identifying community age-friendly service needs
Source: BMC Geriatr. 2026 Mar 25;26:623. doi: 10.1186/s12877-026-07383-0 (PMC13137549; doi:10.1186/s12877-026-07383-0)
Supplement: Supplementary file 1 — Supplementary Material 1. [file 12877_2026_7383_MOESM1_ESM.docx]

**Supplementary File S1**

Detailed Scoring Criteria for individual capability Indicators.

This supplementary file provides detailed definitions of the five-point ordinal scoring criteria (0–4) for each individual capability indicator used in this study. All indicators were assessed based on the individual’s usual performance in daily life.

1. Eating

Ability to bring food to the mouth using appropriate utensils and swallow safely.

Score 4: Independently eats using utensils and swallows safely without choking.

Score 3: Eats independently with verbal guidance or reminders, or independently uses assistive devices, without choking.

Score 2: Requires minor hands-on assistance during eating; occasional choking occurs (≥ once per month).

Score 1: Requires substantial hands-on assistance during eating; frequent choking occurs (≥ once per week).

Score 0: Completely dependent on others for feeding, or has severe swallowing difficulty, or requires tube feeding.

2. Personal Grooming

Includes washing face, brushing teeth, combing hair, shaving, and nail care.

Score 4: Completes all grooming activities independently.

Score 3: Completes grooming with verbal guidance or reminders.

Score 2: Requires assistance but performs most tasks independently.

Score 1: Primarily relies on others but can cooperate.

Score 0: Completely dependent on others and unable to cooperate.

3. Bathing

Ability to wash and dry the body.

Score 4: Bathes independently without assistance.

Score 3: Bathes with verbal guidance or reminders.

Score 2: Requires assistance but completes most bathing tasks independently.

Score 1: Primarily dependent on others but able to cooperate.

Score 0: Completely dependent on others and unable to cooperate.

4. Dressing and Undressing (Upper Body)

Score 4: Independently dresses and undresses the upper body.

Score 3: Completes with verbal guidance or reminders.

Score 2: Requires assistance but performs most tasks independently.

Score 1: Primarily dependent on others but able to cooperate.

Score 0: Completely dependent on others and unable to cooperate.

5. Dressing and Undressing (Lower Body and Footwear)

Score 4: Independently dresses and undresses trousers, shoes, and socks.

Score 3: Completes with verbal guidance or reminders.

Score 2: Requires assistance but performs most tasks independently.

Score 1: Primarily dependent on others but able to cooperate.

Score 0: Completely dependent on others and unable to cooperate.

6. Urinary Continence

Score 4: Full urinary control with normal voiding frequency.

Score 3: Daytime continence preserved; increased nocturia or reduced nocturnal control; independently uses pads or diapers if needed.

Score 2: Mostly continent during the day with occasional incontinence (<1/day but >1/week); requires minor assistance with continence aids.

Score 1: Frequent daytime incontinence (≥1/day) and nocturnal incontinence; requires substantial assistance with continence aids.

Score 0: Complete urinary incontinence or use of an indwelling urinary catheter.

7. Bowel Continence

Score 4: Normal bowel control.

Score 3: Occasional constipation or fecal incontinence (<1/week); independently uses suppositories or pads.

Score 2: Frequent constipation or fecal incontinence (<1/day but >1/week); requires minor assistance.

Score 1: Daily constipation or fecal incontinence (≥1/day); requires substantial assistance.

Score 0: Severe constipation or complete fecal incontinence requiring full assistance for bowel care and skin hygiene.

8. Toileting

Score 4: Independently uses the toilet and performs personal hygiene.

Score 3: Completes toileting with verbal guidance or reminders.

Score 2: Requires assistance but performs most toileting tasks independently.

Score 1: Primarily dependent on others but able to cooperate.

Score 0: Completely dependent on others and unable to cooperate.

9. Bed Mobility

Score 4: Independently turns in bed and moves between lying and sitting.

Score 3: Completes bed mobility with verbal guidance or reminders.

Score 2: Requires assistance but performs most movements independently.

Score 1: Primarily dependent on others but able to cooperate.

Score 0: Completely dependent on others and unable to cooperate.

10. Bed–Chair Transfer

Score 4: Independently transfers between sitting and standing.

Score 3: Completes transfers with verbal guidance or reminders.

Score 2: Requires assistance but performs most transfers independently.

Score 1: Primarily dependent on others but able to cooperate.

Score 0: Completely dependent on others and unable to cooperate.

11. Walking on Level Ground

Score 4: Independently walks approximately 50 m without assistance and without fall risk.

Score 3: Walks approximately 50 m with supervision, guidance, or assistive devices.

Score 2: Requires minor physical assistance while walking.

Score 1: Requires substantial physical assistance while walking.

Score 0: Unable to walk.

12. Stair Climbing

Score 4: Independently climbs ≥15 consecutive steps without assistance.

Score 3: Independently climbs 10–15 steps without assistance.

Score 2: Completes stair climbing with verbal guidance or reminders.

Score 1: Requires assistance but performs most steps independently.

Score 0: Primarily or completely dependent on others for stair use.

13. Time Orientation

Score 4: Oriented to year and month; date or weekday deviation ≤1 day.

Score 3: Mild impairment; confusion regarding year, month, or date (≥2-day deviation).

Score 2: Poor orientation; aware only of season or half-year.

Score 1: Very poor orientation; aware only of day/night or morning/afternoon.

Score 0: No sense of time.

14. Spatial Orientation

Score 4: Independently navigates familiar environments.

Score 3: Unable to go out alone but accurately knows home address.

Score 2: Unable to go out alone but knows partial address information.

Score 1: Knows very limited location information.

Score 0: No spatial orientation.

15. Person Orientation

Score 4: Recognizes familiar individuals and understands relationships.

Score 3: Recognizes most cohabiting individuals.

Score 2: Recognizes some family members or caregivers.

Score 1: Recognizes only self or very few familiar individuals.

Score 0: Recognizes no one, including self.

16. Memory

Score 4: Memory appropriate for age and social functioning.

Score 3: Mild impairment (recalls 0–1 of three words after 5 minutes).

Score 2: Moderate impairment affecting recent memory.

Score 1: Severe impairment affecting remote memory.

Score 0: Complete memory disorganization or inability to recall past events.

17. Comprehension

Score 4: Understands spoken language normally.

Score 3: Understands speech with increased response time.

Score 2: Requires frequent repetition or simplified language.

Score 1: Severe comprehension difficulty requiring substantial assistance.

Score 0: Unable to understand speech.

18. Expression

Score 4: Expresses needs and ideas clearly.

Score 3: Expresses needs with increased time.

Score 2: Expression is difficult and requires frequent prompting.

Score 1: Severe expressive difficulty requiring substantial assistance.

Score 0: Unable to express needs.

19. Aggressive Behavior

Score 4: No aggressive behavior observed.

Score 3: Aggressive behavior occurred within the past 2 months.

Score 2: Aggressive behavior occurred within the past month.

Score 1: Aggressive behavior occurred within the past 2 weeks.

Score 0: Aggressive behavior occurred within the past week.

20. Depressive Symptoms

Score 4: No depressive or negative emotional symptoms observed.

Score 3: Negative emotions within the past 2 months.

Score 2: Negative emotions within the past month.

Score 1: Negative emotions within the past 2 weeks.

Score 0: Negative emotions within the past week.

21. Level of Consciousness

Score 4: Fully alert and appropriately responsive.

Score 3: Drowsy but arousable with verbal or physical stimulation.

Score 2: Confused with impaired attention and orientation.

Score 1: Stuporous or intermittently responsive, or coma.

Score 0: No response to external stimuli.

22. Vision

Score 4: Normal vision under best correction.

Score 3: Able to read large print but not standard text.

Score 2: Limited vision; recognizes objects but not text.

Score 1: Perceives only light, color, or shape.

Score 0: Complete blindness.

23. Hearing

Score 4: Normal hearing.

Score 3: Difficulty hearing soft speech or speech at >2 m.

Score 2: Requires quiet environment or loud speech for communication.

Score 1: Understands speech only when spoken loudly or slowly.

Score 0: Complete deafness.

24. Managing Daily Affairs

Score 4: Independently plans and completes daily tasks.

Score 3: Requires supervision or guidance.

Score 2: Requires minor assistance.

Score 1: Requires substantial assistance.

Score 0: Completely dependent on others.

25. Using Transportation

Score 4: Independently uses bicycle or public transportation.

Score 3: Independently uses public transportation.

Score 2: Independently uses taxis.

Score 1: Uses public transportation only with assistance.

Score 0: Unable to go out independently.

26. Social Interaction

Score 4: Actively participates and interacts appropriately.

Score 3: Adapts to simple environments with mild communication difficulties.

Score 2: Socially withdrawn and passive; vulnerable to deception.

Score 1: Limited interaction with inappropriate expression.

Score 0: Unable to engage in social interaction.

27. Digital Literacy

Score 4: Proficient use of digital devices and applications.

Score 3: Basic use for common tasks (calls, messages, browsing).

Score 2: Limited to very basic operations.

Score 1: Only minimal operations.

Score 0: Unable to use digital devices.

28. Chronic Disease Status

Score 4: 0–1 well-controlled chronic disease without functional impact.

Score 3: 1–2 stable chronic diseases with minimal functional limitation.

Score 2: Multiple chronic diseases with unstable control requiring partial assistance.

Score 1: Multiple poorly controlled diseases with major functional limitations.

Score 0: Severe or uncontrolled disease requiring full-time health management.
