# Supplementary material for: A dual-capability digital portrait framework for identifying community age-friendly service needs
Source: BMC Geriatr. 2026 Mar 25;26:623. doi: 10.1186/s12877-026-07383-0 (PMC13137549; doi:10.1186/s12877-026-07383-0)
Supplement: Supplementary file 2 — Supplementary Material 2. [file 12877_2026_7383_MOESM2_ESM.docx]

Supplementary File S2. Detailed scoring criteria for family caregiving capacity assessment

1. Financial resources

Definition: Ratio of older adults’ per capita monetary assets to local residents’ per capita monetary assets (a).

Scoring:

a < 0.4: 0 points

0.4 ≤ a < 0.8: 1 point

0.8 ≤ a < 1.2: 2 points

1.2 ≤ a < 1.6: 3 points

a ≥ 2.0: 4 points

2. Medical expenditure burden

Definition: Proportion of older adults’ medical expenditure in total household consumption (β).

Scoring:

β < 0.15: 4 points

0.15 ≤ β < 0.25: 3 points

0.25 ≤ β < 0.35: 2 points

0.35 ≤ β < 0.45: 1 point

β ≥ 0.45: 0 points

3. Housing conditions

Definition: Ratio of older adults’ per capita housing area to local residents’ per capita housing area (γ).

Scoring:

γ < 0.4: 0 points

0.4 ≤ γ < 0.8: 1 point

0.8 ≤ γ < 1.2: 2 points

1.2 ≤ γ < 1.6: 3 points

γ ≥ 1.6: 4 points

4. Daily caregiving time

Definition: Average daily caregiving time that family members can provide (T).

Scoring:

T < 0.5 h: 0 points

0.5 ≤ T < 2 h: 1 point

2 ≤ T < 4 h: 2 points

4 ≤ T < 8 h: 3 points

T ≥ 8 h: 4 points

5. Caregiving skills

Definition: Types of caregiving skills possessed by family caregivers.

Scoring components:

Basic caregiving skills: 1.4 points

Medical caregiving skills: 1.4 points

Emergency response skills: 0.6 points

Cognitive care skills: 0.6 points

6. Communication and interaction

Definition: Frequency and duration of effective communication (≥10 minutes per interaction).

Scoring:

Face-to-face/video communication: 0.5 points per interaction per month

Telephone communication: 0.3 points per interaction per month (Maximum score: 3 points)

7. Participation in decision-making

Definition: Assessed based on the “shared decision-making continuum” model.

Scoring:

Autonomous decision-making: 4 points

Shared decision-making: 3 points

Informed consent: 2 points

Passive awareness: 1 point

No awareness: 0 points

8. Family conflict level

Definition: Assessed using a modified Family Conflict Scale.

Scoring (deductive):

Verbal conflict: −0.5 points per occurrence per month

Behavioral confrontation: −1 point per occurrence per month

Prolonged cold conflict: −2 points per occurrence per month
