# Supplementary material for: A dual-capability digital portrait framework for identifying community age-friendly service needs
Source: BMC Geriatr. 2026 Mar 25;26:623. doi: 10.1186/s12877-026-07383-0 (PMC13137549; doi:10.1186/s12877-026-07383-0)
Supplement: Supplementary file 3 — Supplementary Material 3. [file 12877_2026_7383_MOESM3_ESM.docx]

Supplementary Table S1. Individual capability–service need mapping matrix

| **No.** | **Capability** | **Service needs corresponding to capability score** | | | | |
| --- | --- | --- | --- | --- | --- | --- |
|  |  | **Score 4** | **Score 3** | **Score 2** | **Score 1** | **Score 0** |
| 1 | Eating | Anti-slip placemats; heat-resistant tableware; lightweight age-friendly utensils | Eating reminders; meal arrangement assistance; assistive utensils (e.g., anti-tremor or angled spoons) | Minor feeding assistance; swallowing training; soft or semi-liquid diet | Full feeding assistance; swallowing rehabilitation; aspiration prevention care | Feeding services; tube feeding care; nutritional support; aspiration risk management |
| 2 | Personal grooming (washing face, brushing teeth, combing hair) | Age-friendly washing environment; anti-slip mats | Grooming reminders; easy-grip toiletries | Assistance with face washing, hair combing, tooth brushing; basic nail care | Full grooming assistance; skin moisturizing care | Complete grooming care; oral sponge brushing; intensive skin care |
| 3 | Bathing | Anti-slip flooring; grab bars; shower chair; handheld shower | Bathing reminders; optimized placement of bathing supplies | Upper-body/back bathing assistance; anti-slip and thermal protection | Full bathing assistance; safe in-and-out bathing care | Bed bathing; assisted hair washing; pressure ulcer prevention |
| 4 | Dressing/undressing upper body | Age-friendly clothing (Velcro fasteners, large buttons) | Dressing prompts; clothing change reminders | Assistance with buttons/zippers; mild dressing assistance | Full assistance with upper-body dressing/undressing | Full-body dressing care; nursing garments |
| 5 | Dressing/undressing lower body and footwear | Anti-slip easy-to-wear shoes; sock aids | Prompts for putting on trousers/socks; sitting posture guidance | Assistance pulling up trousers; sock aids | Full assistance with trousers and footwear | Bed-based dressing management; comprehensive care |
| 6 | Urinary continence | Night lights; grab bars; age-friendly toileting environment | Night-time toileting reminders; independent management of pads/diapers | Mild incontinence management; timed voiding; pelvic floor muscle training | Extensive incontinence assistance; diaper changes; skin care | Catheter care; perineal hygiene; incontinence-associated dermatitis prevention |
| 7 | Bowel continence | Bowel health management; dietary fiber advice | Occasional constipation/incontinence management; self-use guidance for suppositories | Bowel habit training; assisted use of suppositories/bedpans | Extensive bowel assistance; commode management; skin cleansing and moisturizing | Enema/suppository care; full cleaning assistance; perianal skin protection |
| 8 | Toileting | Grab bars; anti-slip mats; smart toilet | Toileting reminders and safety supervision | Assistance with clothing removal and wiping; sit-to-stand support | Full toileting assistance; transfer care | Bedpan care; excretion cleaning; incontinence management |
| 9 | Bed mobility (turning, sitting up, lying down) | Bedside anti-slip mats; age-friendly bed rails | Verbal prompts for turning/sitting; bedside supervision | Assistance with turning and sitting up; pressure-relief cushions | Full assistance with turning and sitting; transfer protection | Complete passive repositioning; pressure ulcer prevention; repositioning devices |
| 10 | Bed–chair transfer (bed↔chair, sit↔stand) | Age-friendly transfer environment; grab bars; appropriately sized chairs | Verbal guidance; risk reminders; assistive devices if needed | Light support for stability; transfer belts | Full assisted transfers; standing assist devices | Full transfer care; hoists and fall-prevention equipment |
| 11 | Walking on flat ground | Independent walking ~50 m; anti-slip shoes | Supervised walking; cane/walker use; fall prevention reminders | Light arm support; gait training | Extensive support during walking; mobility aids | Wheelchair mobility; bed-based care; passive lower-limb training |
| 12 | Stair climbing | Independent climbing of 10–15 steps; complete handrails | Verbal prompts; close supervision | Light arm support; pace guidance | Extensive support; safety protection | Stair transfer assistance; stair-lift devices if necessary |
| 13 | Time orientation | Time cues (large-font clocks/calendars); regular routines | Verbal date/time prompts; reminder boards | Orientation training; structured daily routines | Circadian rhythm management; one-to-one time prompting | Continuous supervision; simplified cognitive stimulation; safety monitoring |
| 14 | Spatial orientation | Clear environmental signage; familiar routes | Address cue cards; supervised outings | Spatial orientation training; visual cues | Environmental simplification | Anti-wandering management; secured environments; full supervision |
| 15 | Person orientation | Display of familiar photos; stable caregiving team | Name prompts; family photo cue cards | Person recognition training; stable caregiving relationships | Simplified social environment; designated caregiver | Non-verbal reassurance; tactile comfort; familiar environment |
| 16 | Memory function | Memory maintenance activities (reading, photos, conversation) | Immediate memory training; simple repetition | Recent memory aids; structured notebooks | Music therapy; familiar-object stimulation | Full supervision; visual/tactile stimulation |
| 17 | Comprehension ability | Lifelong learning; acquisition of new knowledge | Slower speech; short sentences; extended response time | Simplified language; gestures/pictures; repetition | One-to-one communication; multimodal support | Fully non-verbal communication; emotional reassurance |
| 18 | Expression ability | Normal verbal expression | Patient listening; sufficient response time | Word cue cards; picture boards | Communication boards; gestures; visual symbols | Recognition of non-verbal expressions; observational care |
| 19 | Aggressive behavior | Reduced noise/light stimulation; conflict avoidance | Psychological counseling; emotional support | Behavioral correction and counseling | Enhanced behavioral intervention; medication if needed | Intensive behavioral and pharmacological management |
| 20 | Depressive symptoms | Moderate expectations; exercise; music | Psychological counseling; emotional support | Enhanced emotional support; medication | Intensive psychotherapy and medication | Intensive treatment and close monitoring |
| 21 | Level of consciousness | Moderate exercise; cardiovascular risk prevention | Daily supervision and reminders | Continuous supervision; possible medication | Comprehensive nursing care | Intensive care with vital sign monitoring |
| 22 | Vision | Adequate lighting; regular vision checks | Large-font materials; high contrast; reading lamps | Visual aids; magnifiers | One-to-one guided walking; tactile cues | Full guidance; environmental safety and fall prevention |
| 23 | Hearing | Quiet communication environment; hearing assessment | Increased volume; face-to-face communication | Hearing aid adjustment; written prompts | Loud and slow speech; gestures | Fully visual communication; enhanced non-verbal care |
| 24 | Managing daily affairs | Independent management | Reminders and checks | Mild assistance | Extensive assistance | Full dependence |
| 25 | Use of transportation (A25) | Independent travel for shopping or social activities | Route prompts; accompaniment to transport points | Taxi booking assistance; light accompaniment | Full accompaniment for public transport | Full escort or complete dependence |
| 26 | Social interaction ability (A26) | Normal social participation | Adaptation to simple social settings | Passive social contact; protective supervision | Restricted social participation | One-to-one reassurance |
| 27 | Digital literacy | Skilled independent use of digital devices | Prompted completion of common tasks | Partial delegation of digital tasks | Most digital tasks delegated | Complete reliance on others |
| 28 | Chronic disease status | Stable condition; independent self-management | Occasional reminders; health education | Partial assistance with health management | Extensive health management support | Full health management and professional medical care |

**Note:**

This table illustrates the mapping logic between individual capability levels and corresponding community-based age-friendly service needs. It is intended for methodological illustration and does not represent an exhaustive list of services.
